# Dataset for EEG signals used to detect the effect of coffee consumption on the activation of SSVEP signal

**DOI:** 10.1016/j.dib.2020.105174

**Published:** 2020-01-25

**Authors:** Bikash K. Pradhan, Kishore K. Tarafdar, Suraj K. Nayak, Anwesha Khasnobish, Sumit Chakravarty, Sirsendu S. Ray, Kunal Pal

**Affiliations:** a Department of Biotechnology and Medical Engineering, National Institute of Technology, Rourkela, 769008, India; b TCS Research and Innovation, Kolkata, 700156, India; c Department of Electrical Engineering, Kennesaw State University, Marietta, GA 30060, USA

**Keywords:** EEG, Steady-state visual evoked potential, Caffeinated coffee

## Abstract

A set of electroencephalogram (EEG) data was obtained in the National Institute of Technology, Rourkela, India, from six individuals in the presence of seven photic stimuli of different frequencies (range: 3 Hz–30 Hz). The EEG data were recorded prior to, and post-consumption of caffeinated coffee for detecting the influence of coffee consumption on the initiation of steady-state visual evoked potential (SSVEP) signals in different regions of the brain. The data supports the article: “Data mining-based approach to study the effect of consumption of caffeinated coffee on the generation of steady-state visual evoked potential signals” [1]. The obtained dataset can also be used to have more insight into the brain response during the post-consumption of coffee using different feature extraction, classification, and SSVEP signal detection techniques.

**Table:** Specification Table

Subject	Biomedical signal processing, Neuroscience
Specific subject area	Cognitive neuroscience, Steady-state visual evoked potential, EEG acquisition.
Type of data	Raw data of 22-channel EEG signals and a single-lead electrocardiogram (ECG) signal before and after the stimulus (consumption of caffeinated coffee).
How data were acquired	Data were acquired using a 32-channel EEG acquisition system (RMS Maximus 32 EEG machine)
Data format	Raw data (.mat file)
Parameters for data collection	6 healthy participants (22–28 years age range) were considered for the analysis.
Description of data collection	The EEG signals were acquired prior-consumption as well as post-consumption of caffeinated coffee. The recordings were made by providing photic stimuli of frequencies that lie within the range of 3 Hz–30 Hz. The corresponding recorded signals were used to understand the influence of caffeinated coffee consumption on the SSVEP signal generation.
Data source location	National Institute of Technology, Rourkela, India
Data accessibility	With the article

**Table:** 

**Value of the Data** • The current dataset is recorded in the presence of seven photic stimuli frequencies during before and after-consumption of coffee. The pre-consumption data can be used to analyze the effect of SSVEP stimulus frequencies in the different parts of the brain. • The pre- and post-stimulus data combined can be used to assess the effect of caffeinated coffee on altering the SSVEP signals. • The 22-channel EEG data can be used for frequency-domain analysis and mapping of different brain regions using the Fast Fourier transform algorithm. • The single-lead ECG data can be used to study the impact of caffeinated coffee on heart physiology. • The dataset can also be explored with different feature extraction, classification, and SSVEP detection algorithms.

## 1 Data

**Fig. 1:**
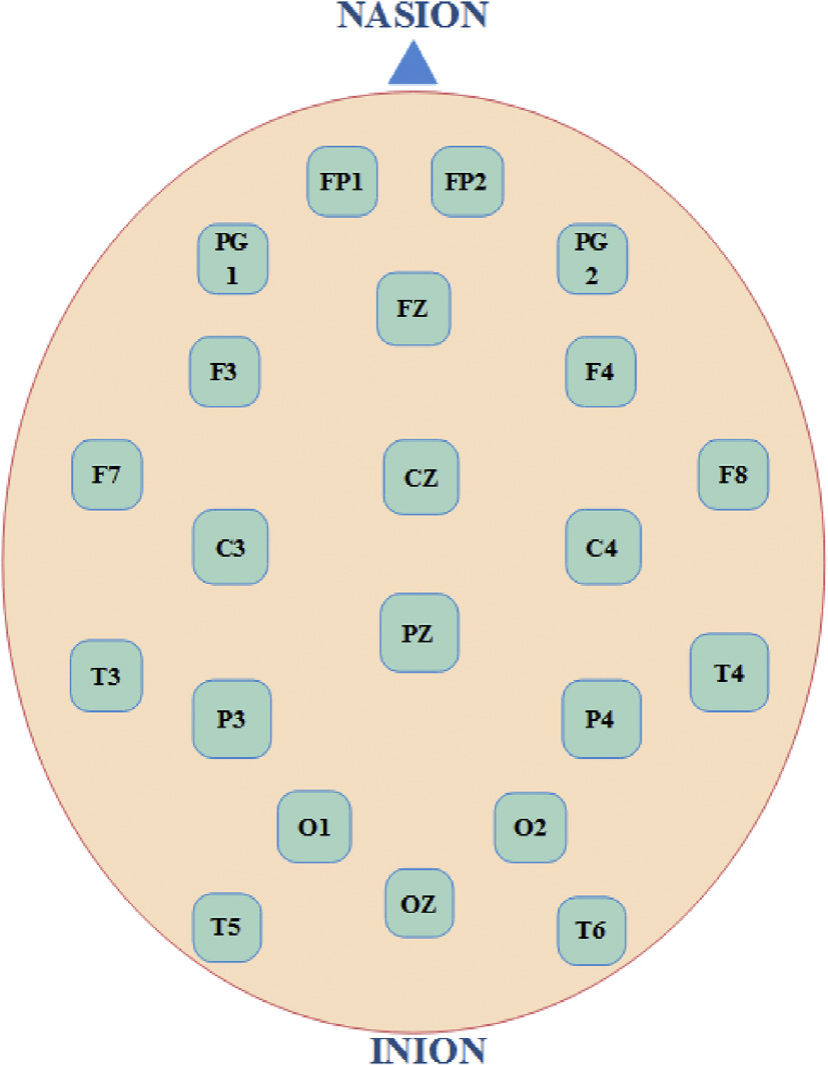
Placement of electrodes on the scalp using 10–20 international standard.

The steady-state visual evoked potential (SSVEP) is generated in the parieto-occipital regions of the brain whenever a source of light of constant stimuli frequency, is focused on the retinal cells [2,3]. Caffeine, an essential constituent of coffee, is believed to have a partial impact on different brain regions such as parietal and occipital cortex areas [4,5]. The current data set can be used to evaluate the post-consumption effect of coffee on the SSVEP activity in the brain regions. The EEG signals were recorded using a clinical EEG acquisition machine (RMS Maximus 32 CH). The equipment has 22 EEG channel electrodes, 2 mastoid electrodes, 1 ground electrode and two limb electrodes for ECG recording. The EEG electrodes were placed on the brain following the 10–20 standard positioning (Fig. 1). The sampling frequency of the recording was maintained at 256 Hz.

**Fig. 2:**
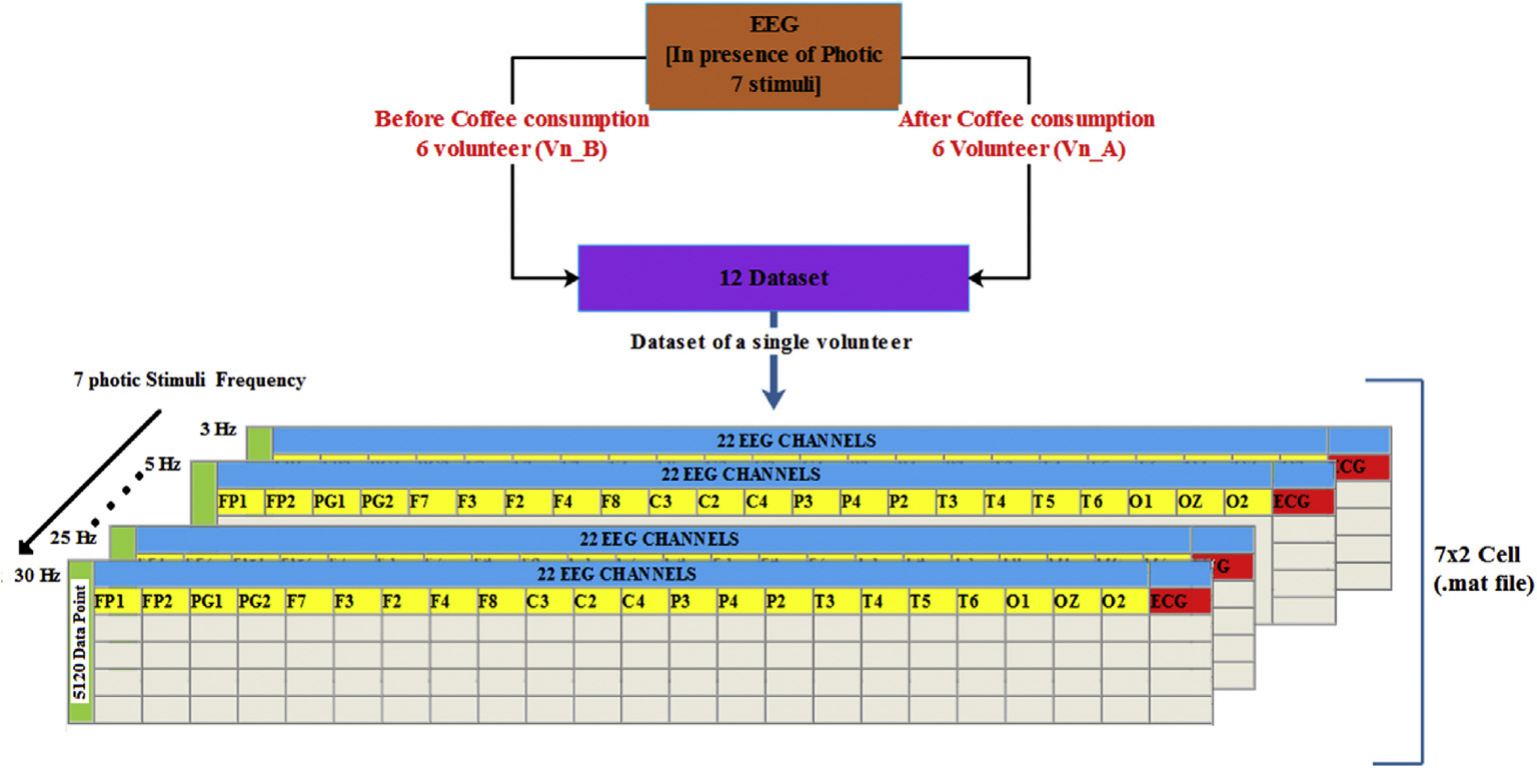
Description of the data (.mat file) of a single recording.

The recorded EEG data from a single volunteer contains the embedded signal of 7 different photic stimulus frequencies of 20s duration and the resting period of 10-sec in between two stimuli frequency. The useful 20s data for each photic stimulus frequency is then extracted [1]. The 20s duration data can be divided into different trials of varying length to increase the sample size as per user requirement. The EEG data of each volunteer was stored in a.mat file, represented in the format of “Vn_B” and “Vn_A”, where n represents the serial number of volunteers, and B and A represent before coffee and after coffee consumption condition, respectively. Information regarding the data collected is presented in Table 1. Each.mat file contains a 7X2 cell array. The first column of the cell array contains the EEG signal in a 5120X23 matrix format, and the 2nd column contains the corresponding photic stimuli frequency. The number of rows in each data matrix is equal to the number of data points (256X20 = 5120) and the first 22 columns contains the corresponding amplitude value of each EEG channel (FP1, FP2, PG1, PG2, F7, F3, FZ, F4, F8, C3, CZ, C4, P3, P4, PZ, T3, T4, T5, T6, O1, O2, and OZ) at that data point. The last column contains the amplitudes of a single lead ECG signal (Fig. 2) during the recording.

**Table 1:** The data of recorded EEG signals.

Volunteer ID	Age (in years)	Sex	Stimulus condition (Caffeinated Coffee)	.mat file
001	23	Male	Before	V1_B
			After	V1_A
002	29	Male	Before	V2_B
			After	V2_A
003	21	Male	Before	V3_B
			After	V3_A
004	25	Male	Before	V4_B
			After	V4_A
005	24	Male	Before	V5_B
			After	V5_A
006	26	Male	Before	V6_B
			After	V6_A

## 2 Experimental design, materials, and method

This experiment is based on a within-group analysis that consists of two stages. In stage 1, EEG signals were acquired in the presence of seven photic stimuli frequency (3 Hz, 5 Hz, 10 Hz, 15 Hz, 20 Hz, 25 Hz, and 30 Hz). In stage 2, EEG signals were recorded 5 min after consumption of caffeinated coffee following the same protocol as in stage 1. Two types of stimuli were used in this experiment. A primary, photic stimulus of a certain frequency (to generate the SSVEP response) and a secondary stimulus, caffeinated coffee (to find the effect of coffee consumption on SSVEP). The stimuli frequency values used in this experiment were chosen in such a way that it will cover all the EEG bands (Table 2) [6].

**Table 2:** List of stimuli frequency considered in the experiment.

Frequency Band	Range of Frequency (in Hz)	Frequency considered in this study (in Hz)
Delta	0–4	3
Theta	4–8	5
Alpha	8–13	10
Low-beta	12.5–16	15
Beta	16.5–20	20
High-beta	20.5–28	25
Low-gamma	30–100	30

**Fig. 3:**
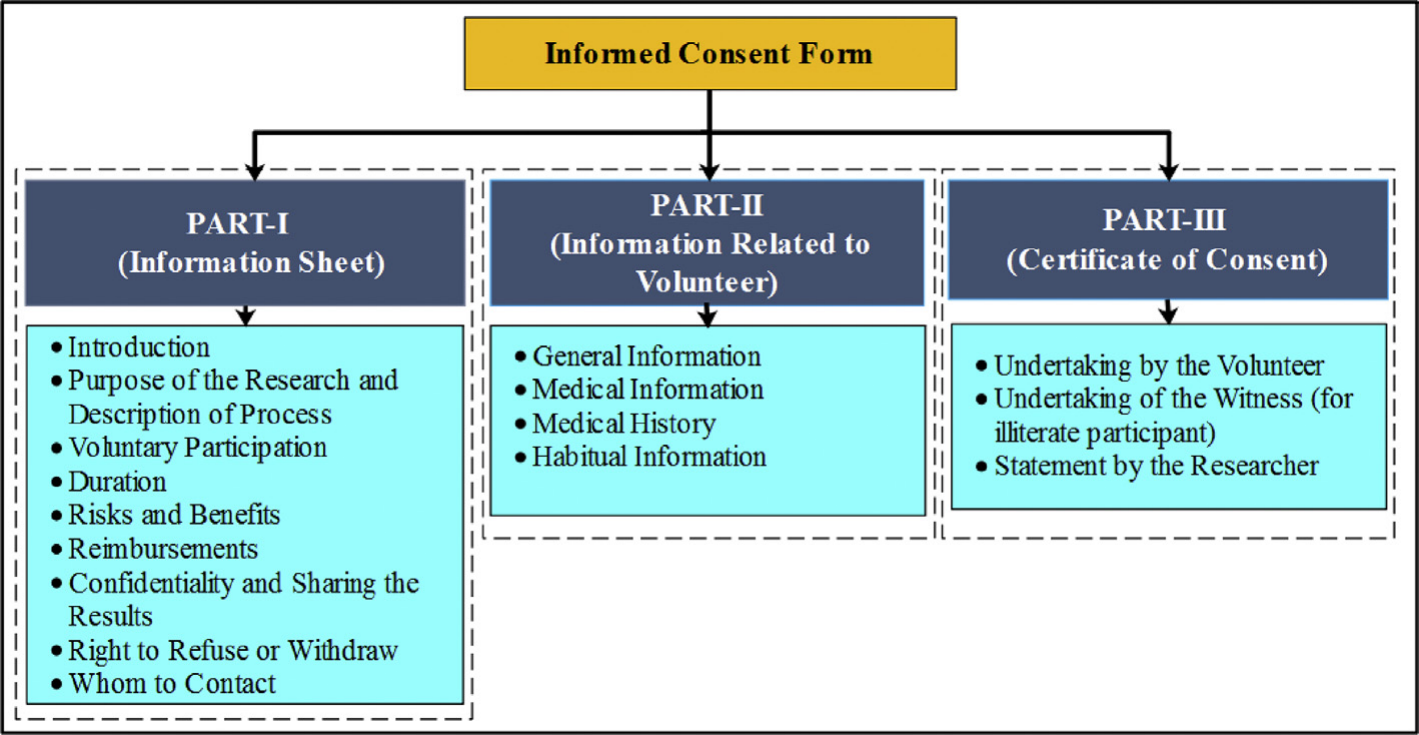
The different subsections of the consent form.

Six healthy male volunteers, who are living a sedentary life and frequent coffee drinkers, were included in this experiment. Prior to recording, permission from the Institute ethical committee (NIT Rourkela) was taken for the recording of the EEG signals vide office order #NITRKL/IEC/FORM/2/35/4/11/001, Dated 13/12/2013. The participants were explained the detailed procedure and purpose of the experiment. Further, they were urged to sign a consent form as a record of agreement of their voluntary participation in the experiment. The consent form was prepared following the guidelines of WHO's (World Health Organisation) informed consent for clinical study as a reference [7] and is divided into three parts. Part-I contains the information related to the research, part-II contains the Information related to the volunteers and part-III contain the declaration made by the participant. A sample consent form has also been attached as a supplementary file. Fig. 3 shows the different sections of the consent form, used in this experiment. The volunteers were also assured that they can leave the experimental procedure at any time if they feel uncomfortable or change their minds.

The EEG signals were recorded using a 32-channel EEG machine, capable of recording 22 EEG signals from different brain regions. The electrodes were placed on the scalp using an electrode cap. The electrodes on the cap were arranged following the 10–20 international standard configurations. After mounting the electrode cap, the contact impedances of the electrodes were adjusted to <20 KΩ. This was achieved by applying a conducting gel on the electrode scalp interface. The sampling frequency of the recording was maintained at 256 Hz. The volunteers were instructed to abstain from food and beverages before 2 h of recording. During recording, they were asked to sit on a chair inside a dim-lit room in a relaxed position. A set of LED was placed in front of their eyes at a distance of 60 cm. The LEDs were made to flicker at seven different frequencies (3 Hz, 5 Hz, 10 Hz, 15 Hz, 20 Hz, 25 Hz, and 30 Hz). The volunteers were requested to stare at the flickering light source during the process of EEG recording. The LED flickered at each stimulus frequency for a period of 20s with a 10s gap between two consecutive frequency stimulation. In the first phase, the EEG signals were recorded without any stimulus (pre-consumption of coffee). In the next phase, the volunteers were served a hot cup of coffee (120 ml containing 1.5 mg of coffee powder). After 5 min of consumption, the EEG signals were re-recorded following the same protocol.

## Conflict of Interest

The authors declare that they have no known competing financial interests or personal relationships that could have appeared to influence the work reported in this paper.
