# Supplementary material for: Dataset for EEG signals used to detect the effect of coffee consumption on the activation of SSVEP signal
Source: Data Brief. 2020 Jan 25;29:105174. doi: 10.1016/j.dib.2020.105174 (PMC7011028; doi:10.1016/j.dib.2020.105174)
Supplement: Multimedia component 2 [file mmc2.pdf]

DEPARTMENT OF BIOTECHNOLOGY & MEDICAL ENGINEERING
NATIONAL INSTITUTE OF TECHNOLOGY, ROURKELA

Informed Consent Form for the Research Project Titled "Understanding the Effect of Coffee on the generation of SSVEP signal"

Title of the Study: Processing and Analysis of EEG Signals to Understand the Effect of Coffee on the generation of SSVEP signal

PART I: Information Sheet

Researchers: Mr. Bikash Kumar Pradhan, Mr. Kishore Tarafdar, and Dr. Kunal Pal
Organization: National Institute of Technology, Rourkela

Introduction

Dr. Kunal Pal is an associate professor in the Department of Biotechnology and Medical Engineering, National Institute of Technology, Rourkela. Mr. Bikash Kumar Pradhan is a research scholar in the same department working under Dr. Pal for his Ph. D. dissertation project.

The researcher is going to provide you information and invite you to be a part of this research. You do not have to decide today whether or not you will participate in the research. Before you decide, you can talk to anyone with whom you are comfortable with about the study. There may be some words that you do not understand. Please feel free to ask the researcher if you have any doubts as you go through the information and the researcher will take time to explain. If you have any questions later, you can ask the same to the researcher anytime you want.

Purpose of the research:

To study the effect of coffee on Autonomic nervous system, respiration and cardiac health.

Type of Research Intervention and Description of Process

The research will involve acquiring EEG at resting condition from healthy volunteers in presence of seven different photic stimuli prior to and post consumption of caffeinated coffee. During the research, each volunteer has to make a visit to the EEG recording station.

Voluntary Participation

Your participation in this research is entirely voluntary. It is your choice whether to participate or not. You may change your mind later and stop participating even if you agreed earlier.

Duration

The research will take place over 3 years in total. During that time, it will be necessary for you to come

once to the ECG recording station. Your EEG signal will be acquired in resting position for 5 minutes.

Risks

There is no known risk associated with this research.

Benefits

Your participation is likely to help the researchers find the answer to the research question. There may not be any benefit to the society at this stage of the research, but future generations are likely to be benefited. The research outcomes may be helpful for future researchers to work in this domain.

Reimbursements

This is a non-funded project, purely for academic purpose. Hence, there are no budget provisions for the reimbursement to the volunteers.

Confidentiality

The information that will be collected from this research project will be kept confidential. Information about you that will be collected during the research will be put away and no-one except the researchers will be able to see it. Any information about you will have an alphanumeric code on it instead of your name. Only the researchers will know what your code is. It will not be shared with or given to anyone except the doctoral scrutiny committee (DSC) nominated/ constituted by the appropriate Institutional authority for the evaluation of the research progress/ thesis examination of the student researcher involved in the study.

Sharing the Results

The results obtained from the analysis of the acquired ECG signals will be used to compile a report, which will lead to the Ph. D thesis dissertation of the student researcher Mr. Bikash Kumar Pradhan. The results will also be used for writing scientific manuscripts and dissemination to the scientific world.

Right to Refuse or Withdraw

You do not have to take part in this research if you do not wish to do so. You may also stop participating in the research at any time you choose. It is your choice and all of your rights will still be respected.

Who to Contact

This project proposal has been reviewed and approved by the Institute Ethical Committee (IEC), of NIT Rourkela, which is a committee whose task is to make sure that research participants are protected from harm. If you wish to find more about the IEC, contact Prof. Krishna Pramanik, Secretary, Institute Ethical Committee, Department of Biotechnology and Medical Engineering, NIT Rourkela

PART II: Information related to the volunteer activities

Volunteer Code:

1. General Information

1. Name (Mr./Ms/Mrs.)____
2. Date Of Birth ____Age_______
3. Address____
   ____
   ____
4. Contact No ______________________e-mail ____
5. Body Weight (kg)__________ Height (mt)__________ BMI (kg/m)_________________

2. Medical information

1. **Are you taking any medication currently? Yes / No**
   *If yes, Please specify the medication*_____________________
   Is the medication steroidal in nature? Yes / No
2. **Medical History**
   a) *None*___________________________
   b) *Specify If Any*___________________
3. **Surgical History**
   a) *None*___________________________
   b) *Specify If Any*___________________
4. **Drug History**
   a) *None* ___________________________
   b) *Specify If Any* ____________________
5. **Sleeping Disorder**
   a) *None*______________________________
   b) *Specify If Any*______________________________
6. **Appetite**
   a) *None*______________________________
   b) *Specify If Any*______________________
7. **Diet Habit**
   a) *Vegetarian*___________________________
   b) *Non-Vegetarian*______________________
   c) *Eggetarian*___________________________
   _

3. Habitual information

1. **Exercise**
   a) **Yes / No**
   b) If Yes, What Type ____
   c) Frequency And Activity
      i) Regularly____
      ii) Occasionally ____
2. **Smoking**
   a) Yes ______________
   b) No ______________
   c) If Yes, Frequency of Smoking
      i) Regularly____
      ii) Occasionally ____

3. Alcohol

a) Yes ______________

b) No ______________

c) If Yes, Frequency of Abuse of Bhang

i) Regularly ____

ii) Occasionally ____

4. Consumption of caffeinated beverages

a) Yes ______________

b) No ______________

c) If Yes, Frequency of Abuse of Bhang

iii) Regularly ____

iv) Occasionally ____

Any other comments: ____

____

____

PART III: Certificate of Consent

I have read the foregoing information, or it has been read to me. I have had the opportunity to ask questions about it and any questions that I have asked have been answered to my satisfaction. I consent voluntarily to participate as a participant in this research.

Name of Participant___________________________
Signature of Participant ___________________________
Date ___________________________
Day/month/year

If illiterate

A literate witness must sign (if possible, this person should be selected by the participant and should have no connection to the research team). Participants who are illiterate should include their thumb-print as well.

I have witnessed the accurate reading of the consent form to the potential participant, and the individual has had the opportunity to ask questions. I confirm that the individual has given consent freely.

Name of witness___________________________ **AND** **Thumb print of participant**
Signature of witness ___________________________
Date ________________________
Day/month/year

Statement by the researcher/person taking consent

I have accurately read out the information sheet to the potential participant, and to the best of my ability made sure that the participant understands that the following will be done:

1. The participant has to make a visit to the EEG recording station.

2. EEG signal will be acquired from the participant in resting position for 5 min.

I confirm that the participant was given an opportunity to ask questions about the study, and all the questions asked by the participant have been answered correctly and to the best of my ability. I confirm that the individual has not been coerced into giving consent, and the consent has been given freely and voluntarily.

A copy of this ICF has been provided to the participant.

Name of Researcher/person taking the consent___________________________

Signature of Researcher/person taking the consent___________________________

Date ___________________________

Day/month/year
